# Towards development of a novel approach for enhancement of TB diagnostic services during the pandemic: A case of primary health care clinics in eThekwini district KwaZulu-Natal: A study protocol

**DOI:** 10.1371/journal.pone.0278305

**Published:** 2022-12-20

**Authors:** Thobeka Dlangalala, Alfred Musekiwa, Tivani Mashamba-Thompson

**Affiliations:** 1 Faculty of Health Sciences, School of Health Systems and Public Health, University of Pretoria, Pretoria, South Africa; 2 Faculty of Health Sciences, University of Pretoria, Pretoria, South Africa; Kasturba Medical College Mangalore, Manipal Academy of Higher Education, INDIA

## Abstract

**Introduction:**

The COVID-19 pandemic has greatly impacted TB diagnostic services in high TB burden settings. This has caused cases to go undetected and increased the number of TB deaths in 2020. Renewed efforts to improve the resilience of TB services during pandemics are required. Therefore, the current study aims to propose a novel approach for conducting TB diagnostic services in high burden settings during the pandemic.

**Methods/Design:**

The proposed study will be conducted in three phases. During the first phase, a geospatial analysis to assess the geographic accessibility of TB diagnostic services will be conducted. In the second phase, the effect of COVID-19 on TB diagnostic services will be determined using an interrupted time series analysis. During the third phase, the barriers and enablers of TB diagnostic services will be explored using patient interviews and a vertical audit. The fourth phase of the study will be guided by the outcomes of the previous three phases where a nominal group technique with key stakeholders will be conducted to propose a novel means for conducting TB diagnostic services during the pandemic. The data of the study will be analyzed using the latest version of ArcGIS, Stata software.

**Discussion:**

The study has received full ethical approval from ethics committees. The results together with input from relevant TB stakeholders will be used to develop a new approach to conducting TB diagnostic services at Primary healthcare clinics.

## Introduction

Tuberculosis (TB) is a communicable disease that up until recently was the leading cause of death by an infectious agent [1]. COVID-19 now occupies the top spot in this regard and its emergence has resulted in devastating consequences for TB control efforts globally. The year 2020 saw newly diagnosed TB cases drop by 18%, from 7.1 million to 5.8 million compared to the previous year [1]. This was due to several reasons including, governments around the globe imposing strict lockdowns to curb the spread of the virus [2, 3]. Furthermore, the emergence of SARS_CoV-2 prompted the diversion of health resources from other diseases to COVID-19 care [4]. Simultaneously, healthcare-seeking behaviors and access to TB services were greatly reduced [4, 5]. Together these factors have led to fewer cases being diagnosed and initiated on treatment which has increased the number of TB deaths for 2020 [1].

South Africa (SA) has one of the highest TB burdens and accounted for 3.3% of the global cases in 2020 [1]. Before COVID-19, the country had made significant strides toward TB control. This was due to the introduction of universal testing and the use of new and repurposed TB drugs [6]. These have allowed the country to meet some key milestones of the end TB strategy set for the year 2020, specifically, reaching a 20% reduction in TB incidence between 2015–2020 [1]. However, due to COVID-19, SA has experienced a similar fate to the rest of the world, reporting a reduction in the number of TB cases notified in 2020 compared to the previous year [1, 7]. A few studies have investigated the impact of COVID-19 on the South African healthcare system with a focus on HIV-related indicators [8, 9]. Some have looked at the impact of COVID-19 on TB services [10, 11]. A study from a district in the Limpopo province showed no significant changes to TB indicators during the first wave of COVID-19 [11]. However, Pillay and colleagues found that TB testing across the country drastically reduced during the national lockdown and has slowly improved after restrictions were lifted, however, as of February 2021 TB testing is still lower than the expected levels [10]. The results imply that COVID-19 has had varying effects on TB services across the country and an investigation into specific regions especially those with a higher TB burden is warranted.

A look at previous epidemics like Ebola and Mers-CoV shows a similar pattern to what is being experienced with COVID-19, where resources and efforts were geared toward fighting the emerging health threat at the expense of existing diseases of importance [12, 13]. This approach has resulted in the indirect cost of such epidemics being greater than the epidemic itself [14]. Modelling studies have predicted a similar outcome for COVID-19 control efforts in light of other health issues like TB [15, 16]. The consequences thereof have already become manifest in high TB burden countries like SA and will likely continue unless effective action is taken [10, 11, 17]. It is important therefore that recovery efforts are not only aimed at restoring TB case detection and treatment levels to those recorded before the COVID-19 pandemic but ensuring that healthcare systems are strengthened so that they remain operational during the current pandemic and other global health crises that may emerge in future. Therefore, our study aims to propose a method for improving TB diagnostic services during COVID-19 at PHC clinics in high burden settings, using eThekwini, SA as a study setting.

A study of this nature is crucial for several reasons, firstly TB is listed among the top ten killers in SA and the leading cause of death among those living with HIV [18]. The main hurdle in eradicating TB in SA is the number of people that become infected that never receive a diagnosis [10]. The reduction in testing experienced at the start of the pandemic suggests that these “missed” people have likely increased [17, 18]. Difficulty in accessing healthcare and reluctance to seek care at the start of COVID-19 are some of the reasons for this [5, 17, 19]. Therefore, it is of the utmost importance that essential services like TB testing are trusted by users and remain in place despite other health challenges arising. Failure to do this will not only undo the strides taken toward TB control in SA but increase the TB burden in coming years. The outcomes of this research can assist in the creation of resilient and robust healthcare systems capable of sustaining essential TB care throughout the different phases of the current pandemic and other health crises that may arise in the future.

## Methods/Design

### Study design

This study will employ an implementation science research approach, which seeks to understand implementation problems for efficacious methods and comes up with evidence-based solutions to improve their uptake and integration into healthcare practice [20]. In implementation research, a variety of study designs are used to address a research question. A scoping review to gain insight into the state of TB services at PHC during COVID-19 has already been conducted and published elsewhere [2]. The findings of the scoping review helped to refine the subsequent objectives. In the first phase of the study, a geospatial analysis will be conducted to determine the accessibility of TB diagnostic services in eThekwini. During the second phase, the impact of COVID-19 on TB diagnostic services in eThekwini will be determined using a quasi-experimental research design. The barriers and facilitators of TB diagnostic services will also be explored through a cross-sectional and a qualitative study design. Once data from the other two phases are analysed, they will be used to guide the nominal group technique (NGT) in the final phase, where key stakeholders will co-create a novel approach for the current TB diagnostic services during COVID-19.

### Conceptual framework

**Fig 1:**
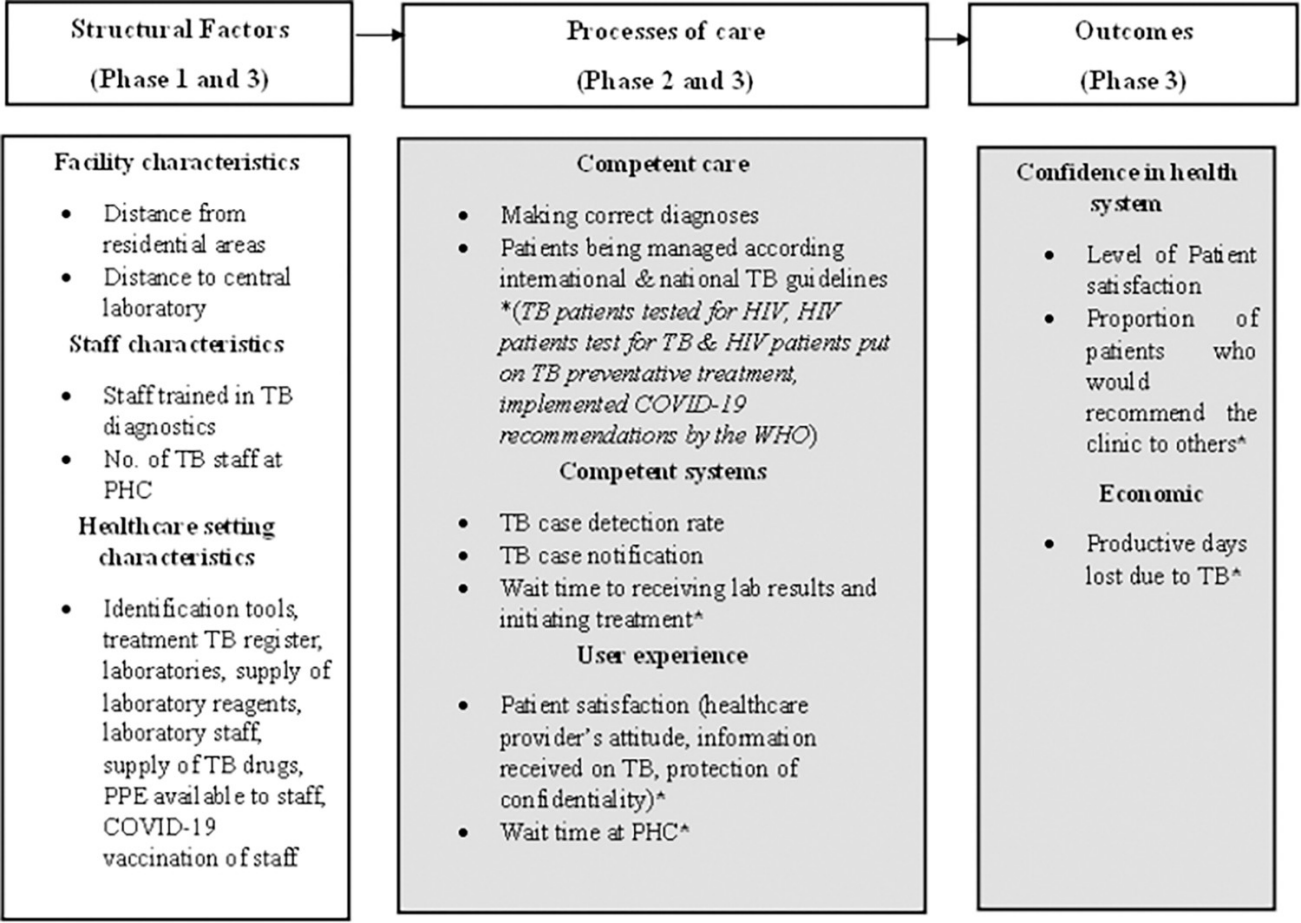
Conceptual framework for measuring quality of TB care for the study: Towards development of a novel approach for enhancement of TB diagnostic services during the pandemic: A case of primary health care clinics in eThekwini district KwaZulu-Natal.

Fig 1 presents the conceptual framework of this study, two frameworks will be combined, Donabedian’s model for assessing the quality of care [21] and the high-quality health system (HQHS) framework by the Lancet commission [22]. Both frameworks assert that high-quality care can improve health outcomes at an individual and population level. Donabedian’s framework states that the quality of healthcare can be measured by looking at the structure, process, and outcome [21]. Structure refers to the physical attributes of the healthcare system such as infrastructure, equipment, number of trained professionals, and the organizational structures in place. The process refers to how care is administered by healthcare personnel and how the users interact with the healthcare system. Lastly, the outcome is the influence of care received on the health status of the patient or population [21]. Each component of the model influences the next, so that good structure will result in good processes which in turn results in good outcomes.

This conceptual framework will be used to guide the first three phases of the study, Fig 1. The quality of care will be assessed using Donabedian’s components of structure, process, and outcome and the quality indicators from the HQHS framework (shaded grey) which have been contextualized for TB care; indicators unique to the HQHS framework have been asterisked. The structure will refer to the included primary healthcare facilities. The study will investigate the accessibility of these facilities and their proximity to the nearest testing laboratory compared to the TB burden in the area. Other structural components assessed will include staff as well as facility characteristics. Processes of care, which according to the HQHS can be divided into competent care, competent systems, and user experience will be measured using the latest evidence-based guidelines [23] and additional measures included by the HQHS framework. The framework also states that high-quality systems should not only improve the health of people but also provide them economic benefits along with increased trust and confidence in health systems [22]. As such, outcome measures for the study will be the confidence in the health system and the economic consequences faced by TB patients. The information derived from this part of the study will then be used to inform the final phase in which a new approach for TB diagnostic services for high-burden settings will be designed by key stakeholders.

### Study setting

The study will be undertaken in the eThekwini district in the province of KwaZulu-Natal (KZN), South Africa. The district is the most densely populated within the province with a population of approximately 3 199 944 [24]. It is a high TB burden district and also has one of the highest cases of COVID-19 in the country. In 2015, it had the highest incidence of TB, totaling 24 588 cases [25]. The district also carries approximately half the country’s burden of extensively drug-resistant TB [26]. A total of 114 public sector clinics provide primary healthcare services in the district comprising 108 fixed clinics and 6 community health centers. The study will be conducted at fixed primary health care clinics; therefore no mobile clinics will be included.

### Phase 1: Geospatial analysis

#### Objective 1: To map the geographic accessibility of TB diagnostics services using geospatial analysis

*Design*. Cross-sectional study.

*Sampling*. All clinics that offer TB testing in the eThekwini district.

*Data source*. Geospatial data of all clinics offering TB diagnostic services and the surrounding residential areas in eThekwini will be obtained from the Department of Health and Geographic positioning system. For the results of the spatial processes to be in “meters” the Universal Transverse Mercator (UTM) zone, 36°S, was applied to all spatial data. Data on the TB prevalence and clinic characteristics will be obtained from the National TB prevalence survey [27] and the DHIS.

*Analysis and mapping*. The coordinates of PHC clinics and residential areas within the eThekwini will be linked to a base map using ArcGIS software. The spatial data of the clinics will be used as inputs to measure proximity to the nearest residential areas. The average speed of 80km/h for the most available and used mode of transport, the minibus taxi, will be applied to estimate the travel time. The detailed model that will be used for this study to determine travel time has been described in a paper by Kuupiel and colleagues [28]. An Autocorrelation tool/Morons index will be used to determine the spatial distribution of PHC clinics in ArcMap. The resulting travel times and distances will be exported into Stata statistical software, where the means and standard deviations will be calculated and recorded. The z scores and p values will also be reported. The results will be used to assess the travel time and distances of TB services compared to the TB prevalence in the surrounding residential areas.

*Outcome measures*. Geographical accessibility in terms of distance and time traveled to PHC clinics that offers TB testing services in eThekwini. The Council for Scientific and Industrial Research (CSIR) has stipulated that social facilities offering public services in South Africa should have a travel distance of 5km to clinics and 30km to hospitals [29]. We will use a travel distance of ≤5km as geographically accessible.

### Phase 2: Quasi-experimental, vertical audit, and qualitative study

#### Objective 2: To determine the effect of the COVID-19 pandemic on TB diagnostic services

*Design*. Regression-based quasi-experimental.

*Sampling*. All PHC clinics providing TB diagnostic services in the eThekwini district will be included.

*Data sources*. Aggregated monthly data from the District of Health Information Systems (DHIS). The time points used will be between January 2018 to January 2022. Any outliers will be identified and corroborated with data from February 2022.

*Analysis*. A segmented linear regression of an interrupted time series analysis will be used to determine the impact of COVID-19 on TB services before and after the start of the pandemic. The outcome variables to be assessed are TB samples sent for testing as well as TB case notifications from PHC clinics in the eThekwini district. The following regression model will be used for the assessment:

$$Y_{t=}\beta_{0}+\beta_{1}T_{t}+\beta_{2}X_{t}+\beta_{3}X_{t}T_{t}+\varepsilon_{t}$$

*Y_t_*: the outcome variable at each time point, number of samples being sent for TB testing, or TB cases notified

*β*_0_: is the baseline level of the outcome (number of samples being sent to the lab and TB cases notified) at the start of the series

*T_t_*: the time passed since the beginning of the study, measured in months.

*X_t_*: is a dummy variable that represents a time before the exposure, COVID-19, and is coded (0). The post-exposure period will be coded (1)

*β_t_*: measures the base trend, how the outcomes change per quarter before COVID-19

*β*_2_: measures the number of samples or cases at the point where (COVID-19) is introduced

*β*_3_: estimates the change in trend following COVID-19

An unchanging slope in the trendline before and after COVID-19 means that the trend in TB samples tested and cases notified were the same despite the emergence of COVID-19. If the slope increases then the outcome variables would have increased after COVID-19 conversely a decreasing slope would indicate a downward trend in the outcome variables. We hypothesize that the trend and the levels will change following the emergence of COVID-19. If *β*_2_ and *β*_3_ produce significant p-values then this would indicate the immediate effect of COVID-19 and its effect over time, respectively. Stata statistical software will be used to conduct this analysis.

*Outcome measures*. The number of TB tests samples sent for testing and the number of TB cases notified.

#### Objective 3: To explore challenges and enablers of TB diagnostics services during the COVID-19 pandemic

*Design*. A clinical audit and a qualitative data gathering technique.

*Sampling*. Two clinics from each of the five sub-districts of eThekwini will be purposively selected. Selection will be purposively determined by the clinic size and the TB burden in the area.

To ascertain user experience and trust in the health system, two patients from each audited facility will be interviewed (S1 File). Only those patients that are 18 years and older and have received a TB service on the day of the audit will be invited for an interview. The interviews will be audio-recorded and conducted using an interview guide. If conceptual saturation is reached before participants at every facility are interviewed then data collection will end. Data saturation will be determined through an iterative process of collecting and analyzing the findings.

*Data sources*. Data will be gathered through an adapted audit checklist developed by the United States Agency for International Development (USAID) for assessing the quality of TB services (S1 File). The responses on the tool will be coded with 1 = yes and 0 = no responses. The final number of yes responses will be divided by the sum of the audit items on the checklist, this will be used to determine the facility score for diagnostic services. The quality of TB diagnostic services will be assessed using three cutoffs, a score of ≥85% will be considered excellent, an average score will be between 50%-84%, and a score of <50% will be considered poor.

For user experience and confidence in the health system, data will be collected through in-depth interviews (S1 File). Before data collection begins both tools will be piloted at two facilities that fall outside of the inclusion criteria. Based on the inputs received from the piloting phase, appropriate changes will be made to the data collection tools.

*Analysis*. The results of the audit will be analyzed to explain the general performance of the PHC facilities and service quality. Descriptive analysis in the form of percentages and counts will be used to describe the outcome variables, and Stata statistical software will be used in this regard.

The results of the interviews will be transcribed and analyzed using thematic content analysis by extracting and grouping similar themes from the patient responses. Direct quotations will also be included if they are found to summarize the main findings of the theme.

*Outcome measures*. Quality of TB diagnostic services.

### Phase 3: Mixed methods—nominal group technique

#### Objective 4: To collaborate with local stakeholders in designing an approach for enhancing the current TB diagnostic services

*Design*. Nominal group technique.

*Sampling*. Key stakeholders will be chosen using criterion-based purposive and a snowballing sampling technique, in the case where a participant cannot take part, snowball sampling will be used to recruit a replacement. For this study, key stakeholders will be defined as individuals that demonstrate knowledge and experience in PHC TB diagnostic services, as well as the TB patients/survivors who use these services. A maximum number of 12 participants are anticipated for the study, this allows for optimal idea generation among group members [30].

*Recruitment strategy*. The eThekwini district health office will be approached to recommend two TB health service researchers or public health specialists who will be able to participate in the nominal group technique, they will be contacted and informed about the purpose of the study. In the case where there are unavailable, they will be asked to recommend someone else who may be suitable to replace them. Similarly, two TB nurses, two TB advocates, and two TB patients will be recruited from a PHC in which the facility audits took place.

*Data source*. Data will be obtained from audio transcripts from the NGT. The workshop will be guided by the findings from the previous objectives. The session will start with the group members introducing themselves and sharing their experience with TB diagnostic services. The PI will then provide context for the workshop and outline the process for the rest of the day. In the first phase of the workshop, the participants will be asked to independently respond to a series of questions posed by the PI regarding the biggest hindrances to TB diagnostics due to COVID-19. Following this, each participant will be allowed to present their answers to the rest of the group. After this stage, the group will clarify and discuss responses after which the ideas will be grouped according to themes. Once data saturation has been reached the ideas will be prioritized from least to most important. A score of one will be given to ideas deemed least important and five will be given to the ideas of most importance. In the second phase of the workshop, all stakeholders will use ranked ideas to come up with improvements to the current TB diagnostic services. The stakeholders with expert knowledge on the workings of TB diagnostic services will suggest ways in which services can be improved while TB patients and survivors will give users insight into the suggestions.

*Analysis*. For the NGT, total scores allocated to each idea during the ranking step will be calculated by adding scores from each participant and assigning the idea an overall score. The proceedings of the NGT will be audio-recorded and transcribed. Qualitative analysis of the five highest-ranking priority themes will be conducted using thematic content analysis. Quality data will be elicited from stakeholders’ presentations which will include motivation for selected themes. During the discussion and clarifying of the priority list, additional data will be collected. This time will also be used to elaborate on the existing themes.

*Outcome measure*. An improved approach to conducting TB diagnostic services at PHC during COVID-19.

### Ethical consideration and data sharing

The study will be conducted following the Protection of Personal Information Act [31]. The participants will be notified about the purposes of the study and allowed to ask questions or withdraw their participation should they choose. Before the start of data collection, eligible participants will be asked to give written consent which will be administered by the primary investigator or a trained research assistant (S1 File). No identifying characteristics or personal information of the participants will be made available at any stage of reporting the results, participants will only be identified by a code or a number. COVID-19 regulations will be closely adhered to according to South Africa’s most recent guidelines. Throughout the study, stakeholders from the PHC clinics, the Department of Health, and members of the community will be engaged to ensure that the cultural dimensions of the community are understood and respected by the research team. Full ethical approval has been received from the University of Pretoria, the Faculty of Health Sciences Research Ethics committee (Reference Number 652/2021), and the KwaZulu-Natal Department of Health (Reference number KZ_202112_012). Once an objective has been completed, the findings will be submitted for publication in a peer-reviewed journal. The results of this study will also be presented at local and international conferences. Upon completion of the study, the findings will also be disseminated among various stakeholders including the department of health and the included primary healthcare clinics.

### Strengths and limitations

To determine the impact of COVID-19 on TB diagnostic services and geographic accessibility the study will use data from all PHC clinics in eThekwini. This gives an accurate depiction of the outcome measures than using a representative sample. Moreover, the power of an interrupted time series analysis, which will be used to determine the impact of COVID-19 on TB diagnostic services, is increased with the use of more data points [32]. Our study will use all the available monthly data since the start of the pandemic to conduct this analysis. Since routine data is used for an interrupted time series analysis, its collection may have been compromised at the start of the COVID-19 pandemic but we will ensure that only reliable data is used. We aim to use all the data collected in the first three objectives of the study to come up with an innovative way of conducting TB diagnostic services. This process will involve TB stakeholders to ensure that the approach is relevant and beneficial for service providers and users alike. This study can offer a foundation for policies aimed at strengthening TB services, which can ultimately help primary healthcare clinics be better prepared for future public health crises. Since the study will only use data from one district, the findings may only be generalizable to settings with a similar TB burden and COVID-19 infection rate as eThekwini.

### Study timeline

The study has not yet begun data collection and participant recruitment. It is anticipated that data cleaning and analysis for the geospatial analysis will commence in September 2022. The interrupted time series analysis will be conducted in October and data collection for the audit and in-depth interviews will be conducted in November and analysed in December of 2022. Following analysis of audit and interview data, the NGT will take place in February of 2022. Participant recruitment for the in-depth interviews will occur on the days that facility audits are conducted. For the Nominal Group Technique, participant recruitment will begin after the completion of the third objective. It is anticipated that data collection will be completed within one year.

## Supporting information

S1 File Informed consent form; audit tool; interview questions. (DOCX)
