# Supplementary material for: Towards development of a novel approach for enhancement of TB diagnostic services during the pandemic: A case of primary health care clinics in eThekwini district KwaZulu-Natal: A study protocol
Source: PLoS One. 2022 Dec 20;17(12):e0278305. doi: 10.1371/journal.pone.0278305 (PMC9767323; doi:10.1371/journal.pone.0278305)
Supplement: S1 File — (DOCX) [file pone.0278305.s001.docx]

**Patient interview consent form**

**PARTICIPANT’S INFORMATION & INFORMED**

**CONSENT DOCUMENT**

**STUDY TITLE: Towards Development of a Novel Approach for Enhancement of TB Diagnostic Services during the Pandemic: A case of Primary Health Care Clinics in eThekwini District KwaZulu-Natal**

**Sponsor: Ninety One UP Skills Bursary**

**Principal Investigators: Thobeka Nomzamo Dlangalala**

**Institution: University of Pretoria**

**DAYTIME AND AFTER HOURS TELEPHONE NUMBER(S):**

**Daytime number/s: 078 915 3494**

**Afterhours number: 078 915 3494**

**DATE AND TIME OF FIRST INFORMED CONSENT DISCUSSION:**

|  |  |  |  | **:** |
| --- | --- | --- | --- | --- |
| **Date** | **month** | **year** |  | **Time** |

**Dear Prospective Participant**

**Dear Mr. / Mrs. .....................................................................................**

**1) INTRODUCTION**

You are invited to volunteer for a research study. I am doing research for a PhD Degree purpose at the University of Pretoria. This information in this document is to help you to decide if you would like to participate. Before you agree to take part in this study you should fully understand what is involved. If you have any questions, which are not fully explained in this document, do not hesitate to ask the researcher. You should not agree to take part unless you are completely happy about all the procedures involved.

**2) THE NATURE AND PURPOSE OF THIS STUDY**

The aim of this study aims to investigate the state of TB diagnostic services at primary healthcare (PHC) clinics during COVID-19. By doing so we wish to find a way to improve TB diagnostic services at primary healthcare clinics during pandemics.

**3) EXPLANATION OF PROCEDURES AND WHAT WILL BE EXPECTED FROM PARTICIPANTS.**

This study involves answering some questions regarding your experience with the TB services that you received.

**4) POSSIBLE RISKS AND DISCOMFORTS INVOLVED**

There are no medical risks associated with the study. The only possible risk and discomfort involved is sharing any negative experiences about the health facility that you regularly attend. You can decline to answer any questions that cause you discomfort.

**5) POSSIBLE BENEFITS OF THIS STUDY**

Although you may not benefit directly. The study results may help us to improve the TB services at primary healthcare clinics.

**6) COMPENSATION**

You will not be paid to take part in the study. There are no costs involved for you to be part of the study.

**7) YOUR RIGHTS AS A RESEARCH PARTICIPANT**

Your participation in this trial is entirely voluntary and you can refuse to participate or stop at any time without stating any reason. Your withdrawal will not affect your access to other medical care.

**8) ETHICS APPROVAL**

This Protocol was submitted to the Faculty of Health Sciences Research Ethics Committee, University of Pretoria, telephone numbers 012 356 3084 / 012 356 3085 and written approval has been granted by that committee (Approval Number…652/2021…………………). The study was also submitted to the Health Research and Knowledge Management Unit of the KZN department of health, telephone number 033 395 2046 and has received written ethical approval (Approval Number… KZ_202112_012….). The study has been structured in accordance with the Declaration of Helsinki (last update: October 2013), which deals with the recommendations guiding doctors in biomedical research involving human/subjects. A copy of the Declaration may be obtained from the investigator should you wish to review it.

**9) INFORMATION**

If I have any questions concerning this study, I should contact:

Dr Tel: (+27)

**10) CONFIDENTIALITY**

All information obtained during the course of this study will be regarded as confidential. Each participant that is taking part will be provided with an alphanumeric coded number e.g. A001. This will ensure confidentiality of information so collected. Only the researcher will be able to identify you as participant. Results will be published or presented in such a fashion that patients remain unidentifiable. The hard copies of all your records will be kept in a locked facility at the Department of Public Health, The University of Pretoria.

**11) CONSENT TO PARTICIPATE IN THIS STUDY**

- I confirm that the person requesting my consent for my child to take part in this study has told me about the nature and process, any risks or discomforts, and the benefits of the study.
- I have also received, read and understood the above written information about the study.
- I have had adequate time to ask questions and I have no objections to participate in this study.
- I am aware that the information obtained in the study, including personal details, will be anonymously processed and presented in the reporting of results.
- I understand that I will not be penalised in any way should I wish to discontinue with the study and that withdrawal will not affect my further treatments.
- I am participating willingly.
- I have received a signed copy of this informed consent agreement.

__________________________________ ________________________

Participant’s name (Please print) Date

__________________________________ ________________________

Participant’s signature Date

__________________________________ ________________________

Researcher’s name (Please print) Date

__________________________________ ________________________

Researcher’s signature Date

AFFIRMATION OF INFORMED CONSENT BY AN ILLITERATE PARTICIPANT

(If suitable)

I, the undersigned, ………………………………………..…, have read and have explained fully to the participant, named ………………………… , the informed consent document, which describes the nature and purpose of the study in which I have asked the him/her to participate. The explanation I have given has mentioned both the possible risks and benefits of the study. The participant indicated that he/she understands that he/she will be free to withdraw from the study at any time for any reason and without jeopardizing his/her standard care.

I hereby certify that the patient has agreed to participate in this study.

___________________________ ________________________

Participant’s name (Please print) Date

__________________________________ ________________________

Participant’s signature Date

__________________________________ ________________________

Investigator's Name (Please print) Date

__________________________________ ________________________

Investigator's Signature Date

__________________________________ ________________________

Name of the person who witnessed

the informed consent (Please print) Date

__________________________________ ________________________

Signature of the Witness Date

**Nominal Group Technique informed consent form**

**PARTICIPANT’S INFORMATION & INFORMED**

**CONSENT DOCUMENT**

**STUDY TITLE: Towards Development of a Novel Approach for Enhancement of TB Diagnostic Services during the Pandemic: A case of Primary Health Care Clinics in eThekwini District KwaZulu-Natal**

**Sponsor: Ninety One UP Skills Bursary**

**Principal Investigators: Thobeka Nomzamo Dlangalala**

**Institution: University of Pretoria**

**DAYTIME AND AFTER HOURS TELEPHONE NUMBER(S):**

**Daytime number/s: 078 915 3494**

**Afterhours number: 078 915 3494**

**DATE AND TIME OF FIRST INFORMED CONSENT DISCUSSION:**

|  |  |  |  | **:** |
| --- | --- | --- | --- | --- |
| **Date** | **month** | **year** |  | **Time** |

**Dear Prospective Participant**

**Dear Mr. / Mrs. .....................................................................................**

**1) INTRODUCTION**

You are invited to volunteer for a research study. I am doing research for a PhD Degree purpose at the University of Pretoria. This information in this document is to help you to decide if you would like to participate. Before you agree to take part in this study you should fully understand what is involved. If you have any questions, which are not fully explained in this document, do not hesitate to ask the researcher. You should not agree to take part unless you are completely happy about all the procedures involved.

**2) THE NATURE AND PURPOSE OF THIS STUDY**

The aim of this study aims to investigate the state of TB diagnostic services at primary healthcare (PHC) clinics during COVID-19. By doing so we wish to find a way to improve TB diagnostic services at primary healthcare clinics during pandemics.

**3) EXPLANATION OF PROCEDURES AND WHAT WILL BE EXPECTED FROM PARTICIPANTS.**

The study is expected to enroll at least 8 participants. Participants will be expected to participate in a workshop where they will be involved in co-designing strategies for improving TB diagnostic services at PHC. The duration of your participation if you choose to enroll and remain in the study is expected to be no more than six and a half hours.

**4) POSSIBLE RISKS AND DISCOMFORTS INVOLVED**

There are no medical risks associated with the study. The only possible risk and discomfort involved is the length of time involved in participating in the workshop. You can decide to with draw participation at any time during the workshop.

**5) POSSIBLE BENEFITS OF THIS STUDY**

Although you may not benefit directly, the study will help generate data that will help in developing a novel approach for conducting TB diagnostic services at PHC clinics during pandemics.

**6) COMPENSATION**

You will not be paid to take part in the study. However, you will be reimbursed for transport

expenses and any other cost that you incur that is directly linked to your participation in this study,

**7) YOUR RIGHTS AS A RESEARCH PARTICIPANT**

Your participation in this workshop is entirely voluntary and you can refuse to participate or stop at any time without stating any reason. Your withdrawal will not affect your access to other medical care.

**8) ETHICS APPROVAL**

This Protocol was submitted to the Faculty of Health Sciences Research Ethics Committee, University of Pretoria, telephone numbers 012 356 3084 / 012 356 3085 and written approval has been granted by that committee (Approval Number…652/2021.). The study was also submitted to the Health Research and Knowledge Management Unit of the KZN department of health, telephone number 033 395 2046 and has received written ethical approval (Approval Number… KZ_202112_012.). The study has been structured in accordance with the Declaration of Helsinki (last update: October 2013), which deals with the recommendations guiding doctors in biomedical research involving human/subjects. A copy of the Declaration may be obtained from the investigator should you wish to review it.

**9) INFORMATION**

If I have any questions concerning this study, I should contact:

**10) CONFIDENTIALITY**

All information obtained during the course of this study will be regarded as confidential. Each participant that is taking part will be provided with an alphanumeric coded number e.g. A001. This will ensure confidentiality of information so collected. Only the researcher will be able to identify you as participant. Results will be published or presented in such a fashion that patients remain unidentifiable. The hard copies of all your records will be kept in a locked facility at the Department of Public Health, The University of Pretoria.

**11) CONSENT TO PARTICIPATE IN THIS STUDY**

- I confirm that the person requesting my consent for my child to take part in this study has told me about the nature and process, any risks or discomforts, and the benefits of the study.
- I have also received, read and understood the above written information about the study.
- I have had adequate time to ask questions and I have no objections to participate in this study.
- I am aware that the information obtained in the study, including personal details, will be anonymously processed and presented in the reporting of results.
- I understand that I will not be penalised in any way should I wish to discontinue with the study and that withdrawal will not affect my further treatments.
- I am participating willingly.
- I have received a signed copy of this informed consent agreement.

__________________________________ ________________________

Participant’s name (Please print) Date

__________________________________ ________________________

Participant’s signature Date

__________________________________ ________________________

Researcher’s name (Please print) Date

__________________________________ ________________________

Researcher’s signature Date

AFFIRMATION OF INFORMED CONSENT BY AN ILLITERATE PARTICIPANT

(If suitable)

I, the undersigned, ………………………………………..…, have read and have explained fully to the participant, named ………………………… , the informed consent document, which describes the nature and purpose of the study in which I have asked the him/her to participate. The explanation I have given has mentioned both the possible risks and benefits of the study. The participant indicated that he/she understands that he/she will be free to withdraw from the study at any time for any reason and without jeopardizing his/her standard care.

I hereby certify that the patient has agreed to participate in this study.

___________________________ ________________________

Participant’s name (Please print) Date

__________________________________ ________________________

Participant’s signature Date

__________________________________ ________________________

Investigator's Name (Please print) Date

__________________________________ ________________________

Investigator's Signature Date

__________________________________ ________________________

Name of the person who witnessed

the informed consent (Please print) Date

__________________________________ ________________________

Signature of the Witness Date

**Interview guide (English)**

**Interview guide**

The interview will begin with the interviewer introducing themselves, briefly stating the reasons for the interview, and reminding the participants of their rights.

**Question 1**

How long did it take you to receive assistance today?

Probe

- Does the wait time influence your health-seeking behaviors?

**Question 2**

How was the attitude of the healthcare provider who assisted you today?

Probe

- Did they treat you with respect?
- Do you believe that your confidentiality was respected?

**Question 3**

Did your healthcare provider give you information on TB?

Probe

- Did they provide the information in a way you understand?
- Did they allow asking questions?
- Did you have the time to discuss your health needs?

**Question 4**

Were you satisfied with the service you received at the clinic today?

- If not, what could’ve improved your experience?

**Question 5**

What do you think should be done to improve the service at this clinic?

**Question 6**

Do you attend this health facility by choice, given the opportunity would you seek care elsewhere?

- Why?
- If answer yes for second part, where would you go instead and why?

**Question 7**

Out of ten, how would you rate the service you received today, with one being the lowest and ten being the highest?

**Interview guide in isiZulu**

Inhlolokhono izoqala ngomhloli ezasisa ngaye, echaze kafushane ngezizathu zenhlolokhono egcine ngokukhumbuza abanganeli inhlolokhono ngamalungelo abo.

**Umbuzo 1**

Kuthathe isikhathi esingakanani ukuthola usizo namhlanje?

(Probe)

Isikhathi osilindayo emtholampilo sinamthalela muni enqubweni yokulandelela ezempilo yakho na?

**Umbuzo 2**

Ukuphathe kanjani umhlengikazi/umhlengi okusizile namhlanje?

(Probe)

Ukuphathe ngenhlonipho yini?

Uyakholelwa ektheni imfihlo ngesifo sakho basihloniphile?

**Umbuzo 3**

Umhlengikazi ukunikile yini imininingwane ngeTB?

(Probe)

Bakuchazele ngendlela oyiqondayo yini?

Bakunikile yini ithuba lokubuza imbuzo?

Ubunaso yini isikhathi sokuxoxa ngezidingo zakho zempilo?

**Umbuzo 4**

Unelisekile ngempatho oyithole emtholampilo namhlanje?

(Probe)

Bawunganelisekile yini engaxazulula leyonkinga?

**Umbuzo 5**

Ngokwakho yini engeziwa ukuphucula impatho yezi guli kulomtholampilo?

**Umbuzo 6**

Usebenzisa lomtholampilo ngokuzithandela wena noma ungayakwenya indawao ngokuzikhethela kwakho?

**Umbuzo 7**

Emaphuzwini ayishumi, ungalibeka kuliphi izinga lomtholampilo, u-1 izinga eliphansi kakhulu bese u-10 izinga eliphezulu?

**Facility Audit tool**

| **Facility Identification** | | | | | | | | | | | | | | | | | | | | | | | | | | |  |
| --- | --- | --- | --- | --- | --- | --- | --- | --- | --- | --- | --- | --- | --- | --- | --- | --- | --- | --- | --- | --- | --- | --- | --- | --- | --- | --- | --- |
| Facility Name |  | | | | | | | | | | | | | | | | | | | | | | | | | |  |
| Location of facility |  | | | | | | | | | | | | | | | | | | | | | | | | | |  |
| **Facility Characteristics** | | | | | | | | | | | | | | | | | | | | | | | | | | |  |
| Locality of facility | Urban  Peri-urban  Rural | | | | | | | | | | | | | | | | | | | | | | | | | |  |
| TB service availability | \| Outpatient only  Inpatient only  Both inpatient and outpatient \| \| --- \| | | | | | | | | | | | | | | | | | | | | | | | | | |  |
| **Facility Visit** | | | | | | | | | | | | | | | | | | | | | | | | | | |  |
| Visit Date | | Interviewer ID and Name | | | Result | | | | | | | | | | | | | | | | | | | | | |  |
| ___/___/______ | | Name: | | | Completed  Partially completed  Respondent unavailable  Facility refused  Postponed  Other (specify)  __________________________ | | | | | | | | | | | | | | | | | | | | | |  |
| Survey start time  [Use the 24-hour clock system, e.g. 14:30] | | Visit 1: | | | Visit 2: (If needed) | | | | | | | | | | | | | | | | | | | | | |  |
| **Availability of services** | | | | | | | | | | | | | | | | | | | | | | | | | | |  |
|  | |  | | **Yes** | **No** | | | | | | | | | | | | | | | | | | | | | |  |
| 1.1 | | Does this facility provide TB screening and diagnosis services? | |  |  | | | | | | | | | | | | | | | | | | | | | |  |
| 1.1.1 | | **[ASK ONLY IF 1.1=YES]**  Does this facility provide those services to children? | |  |  | | | | | | | | | | | | | | | | | | | | | |  |
| 1.2 | | Does this facility provide TB care and treatment services? | |  |  | | | | | | | | | | | | | | | | | | | | | |  |
| 1.2.1 | | **[ASK ONLY IF 1.2=YES]**  Does this facility provide those services to children? | |  |  | | | | | | | | | | | | | | | | | | | | | |  |
|  | | | | | | | | | | | | | | | | | | | | | | | | | | |  |
| 1.3 | Typically, how many days per week are TB-related services offered? | | | |  | | | | | | | | | | | | | | | | | | | | | |  |
| 1.4 | How many service points *(i.e. number of places within the facility)* deliver TB-related services in the facility? | | | |  | | | | | | | | | | | | | | | | | | | | | |  |
| 1.5 | Approximately, how many years do you think TB-related services have been available at this facility? [IF LESS THAN A YEAR, RECORD “00”] | | | |  | | | | | | | | | | | | | | | | | | | | | |  |
| 1.6 | Now, I will ask if the facility provides certain TB-related services. For each service, I want to know whether this facility ever offered the service. For those you answer yes, I will then ask if they were available in the past 12 months. | | | | | | | | | | | | | | | | | | | | | | | | | |  |
|  | | | Services | | (a) Ever provided the service at the facility | | | | | | | | | | | | **[ASK ONLY IF (a)=YES]**  (b) Available at all times in last 12 months | | | | | | | | | |  |
|  |  |  |  |  | **Y** | **N** | **[NR]** | | | | **Yes, available for 12 or more months** | | | | | | | | | | | | | **No, available for <12 months** | | | |
|  | | |  | |  |  |  | | | |  | | | | | | | | | | | | |  | | | |
| 1.6.1 | | | **[ASK ONLY IF 1.1=YES]**  Diagnosis of tuberculosis based on any type of specimen testing (smear, culture, rapid test) | |  |  |  | | | |  | | | | | | | | | | | | |  | | | |
| 1.6.2 | | | **[ASK ONLY IF 1.1=YES]**  Diagnosis of tuberculosis based on clinical symptoms | |  |  |  | | | |  | | | | | | | | | | | | |  | | | |
| 1.6.3 | | | **[ASK ONLY IF 1.2=YES]**  TB medicines given directly to patient by health provider | |  |  |  | | | |  | | | | | | | | | | | | |  | | | |
| **Availability** | | | | | | | | | | | | | | | | | | | | | | | | | | |  |
| 1.6.9 | | | Phone calls to TB patients, e.g. if they miss an appointment, to schedule a home visit, etc. | |  |  |  | | | |  | | | | | | | | | | | | |  | | | |
| 1.6.10 | | | HIV testing and counseling for TB patients | |  |  |  | | | |  | | | | | | | | | | | | |  | | | |
| 1.6.11 | | | Preventive treatment for TB infection (INH + Pyridoxine) | |  |  |  | | | |  | | | | | | | | | | | | |  | | | |
| 1.6.12 | | | Screening of HIV+ patients for TB disease | |  |  |  | | | |  | | | | | | | | | | | | |  | | | |
| 1.6.13 | | | ART for TB/HIV co-infected patients | |  |  |  | | | |  | | | | | | | | | | | | |  | | | |
| 1.6.14 | | | CPT for TB/HIV co-infected patients | |  |  |  | | | |  | | | | | | | | | | | | |  | | | |
| 1.6.15 | | | Viral load testing for TB/HIV co-infected patients | |  |  |  | | | |  | | | | | | | | | | | | |  | | | |
| 1.6.16.1 | | | Diagnosis of MDR-TB | |  |  |  | | | |  | | | | | | | | | | | | |  | | | |
|  | | | **[ASK ONLY IF 1.1=YES]** | | | | | **Y** | | | | | | | | | | | **N** | | | | | | | **[NR]** |  |
| 1.7 | | | Does the facility conduct TB diagnostic tests onsite? | | | | |  | | | | | | | | | | |  | | | | | | |  |  |
|  | | | **[ASK ONLY IF 1.7=YES]**  Do you use the following methods for diagnosing TB in this facility? | | | | | **Y** | | | | | | | | | | | **N** | | | | | | | **[NR]** |  |
| 1.7.1 | | | Clinical signs and symptoms | | | | |  | | | | | | | | | | |  | | | | | | |  |  |
| 1.7.2 | | | Sputum smear microscopy examination – light microscope | | | | |  | | | | | | | | | | |  | | | | | | |  |  |
| 1.7.3 | | | Sputum smear microscopy examination – LED microscope | | | | |  | | | | | | | | | | |  | | | | | | |  |  |
| 1.7.4 | | | Culture – solid media | | | | |  | | | | | | | | | | |  | | | | | | |  |  |
| 1.7.5 | | | Liquid culture (ex, MGIT) | | | | |  | | | | | | | | | | |  | | | | | | |  |  |
| 1.7.6 | | | GeneXpert MTB/RIF based in facility | | | | |  | | | | | | | | | | |  | | | | | | |  |  |
| 1.7.7 | | | GeneXpert OMNI | | | | |  | | | | | | | | | | |  | | | | | | |  |  |
| 1.7.8 | | | Lateral flow urine lipoarabinomannan assay (LF-LAM) | | | | |  | | | | | | | | | | |  | | | | | | |  |  |
| 1.7.9 | | | Chest x-ray | | | | |  | | | | | | | | | | |  | | | | | | |  |  |
| 1.7.10 | | | Access to line-probe assays (LPAs) | | | | |  | | | | | | | | | | |  | | | | | | |  |  |
| 1.7.11 | | | Access to digital CXR | | | | |  | | | | | | | | | | |  | | | | | | |  |  |
| 2. | | | ***Diagnostics Capacity and Testing* [TB Focal Person or Lab Personnel]** | | | | | | | | | | | | | | | | | | | | | | | |  |
|  | | |  | | | | |  | | | | | | | | | | |  | | | | | | |  |  |
| 2.1 | | | Does the facility perform the Amplified Mycobacterium TB Direct Test (MTD)? | | | | |  | | | | | | | | | | |  | | | | | | |  |  |
| 2.2 | | | Does the facility send specimens outside of the facility for TB testing? | | | | |  | | | | | | | | | | |  | | | | | | |  |  |
| 2.3 | | | Does the facility keep records of results of sputum tests? | | | | |  | | | | | | | | | | |  | | | | | | |  |  |
| 2.4 | | | Does the facility have an existing system for quality control (either internal or external) for the specimens assessed in this facility? | | | | |  | | | | | | | | | | |  | | | | | | |  |  |
| 2.5 | | | Does the facility keep records of the results from the quality control (internal or external) procedures? | | | | |  | | | | | | | | | | |  | | | | | | |  |  |
|  | | |  |  | | | | | | | | | | | | | | | | | | | | | |  |  |
| 2.6 | | | **[ASK ONLY IF 2.4=YES]**  Type of quality control practice followed by the facility | None…………………………………….……...………….  Internal quality control only…….………..…….  External quality control only……………..…….  Both internal and external quality control. | | | | | | | | | | | | | | | | | | | | | |  |  |
| 2.6.1 | | | **[ASK ONLY IF 2.6>0]**  How often does the facility perform quality control? | Rarely…………………….…………….……...………….  Sometimes………………………..…….………..…….  Always……………..……………………………………… | | | | | | | | | | | | | | | | | | | | | |  |  |
| 2.7 | | | **[ASK ONLY IF 1.7=YES]**  What type of TB test services are performed at the facility? | | | | | **Y** | | | | | | | | | | | **N** | | | | | | | **[NR]** |  |
| 2.7.1 | | | Ziehl-Neelsen testing for TB (AFB) | | | | |  | | | | | | | | | | |  | | | | | | |  |  |
| 2.7.2 | | | Xpert® MTB/RIF diagnostic testing for TB | | | | |  | | | | | | | | | | |  | | | | | | |  |  |
| 2.7.3 | | | Were there any stock-outs of the diagnostic test supplies in the past 6 months? | | | | |  | | | | | | | | | | |  | | | | | | |  |  |
| \| 3. \| ***Policies, Protocols, and Guidelines* [In-charge or TB Focal Person]** \| \| --- \| --- \| | | | | | | | | | | | | | | | | | | | | | | | | | | |  |
|  | | | Next, I’d like to assess the availability of copies of approved and required protocols, policies, and messages on TB information available at the facility: | | **Observed** | | | **Reported, not observed** | | | | | | | | | | | **Not available** | | | | | | | **[NR]** |  |
| 3.1 | | | The national TB management and control guidelines | |  | | |  | | | | | | | | | | |  | | | | | | |  |  |
| 3.2 | | | **[ASK ONLY IF 2.6>0]**  Guidelines and procedures for quality control (either internal or external) for the specimens assessed in this facility | |  | | |  | | | | | | | | | | |  | | | | | | |  |  |
| 3.3 | | | The National Guidelines for clinical management of TB/ HIV related conditions | |  | | |  | | | | | | | | | | |  | | | | | | |  |  |
| 3.4 | | | **[ASK ONLY IF 1.2=YES]**  Protocol or guideline of essential drug list or essential medicines list | |  | | |  | | | | | | | | | | |  | | | | | | |  |  |
| 3.5 | | | **[ASK ONLY IF 1.2=YES]**  A training manual for DOT providers or volunteers | |  | | |  | | | | | | | | | | |  | | | | | | |  |  |
| 3.6 | | | **[ASK ONLY IF 1.2=YES]**  Guidelines related to MDR-TB diagnosis and treatment (or identification of need for referral) | |  | | |  | | | | | | | | | | |  | | | | | | |  |  |
| 3.7 | | | **[ASK ONLY IF 1.1=YES]**  Flowcharts or algorithms on TB screening | |  | | |  | | | | | | | | | | |  | | | | | | |  |  |
| 3.8 | | | **[ASK ONLY IF 1.1=YES]**  Flowcharts or algorithms on TB diagnosis | |  | | |  | | | | | | | | | | |  | | | | | | |  |  |
| 3.9 | | | TB posters on walls, leaflets, brochures, and pamphlets in local languages for distribution, i.e. educational materials about TB available | |  | | |  | | | | | | | | | | |  | | | | | | |  |  |
| 4. | | | ***Management and Staff* [In-charge or TB Focal Person]** | | | | | | | | | | | | | | | | | | | | | | | |  |
|  | | | Did any providers of TB services at this facility receive new or refresher training in the following topics in the last 24 months? | | **Within 24 months** | | | **Over 24 months** | | | | | | | | | | | **No trained staff** | | | | | | | **[NR]** |  |
| 4.1 | | | Management of TB-HIV co-infection | |  | | |  | | | | | | | | | | |  | | | | | | |  |  |
| 4.2 | | | TB infection control | |  | | |  | | | | | | | | | | |  | | | | | | |  |  |
| 4.3 | | | Diagnosis of tuberculosis using Xpert MTB/RIF | |  | | |  | | | | | | | | | | |  | | | | | | |  |  |
| 4.4 | | | Diagnosis of tuberculosis based on clinical symptoms and/or examination | |  | | |  | | | | | | | | | | |  | | | | | | |  |  |
| 5. | | | \| ***TB Information and Services* [In-charge or TB Focal Person]** \| \| --- \| | | | | | | | | | | | | | | | | | | | | | | | |  |
|  | | | Do staff or personnel provide the following information to TB patients? | | | | | **Y** | | | | | | | | | | | **N** | | | | | | | **[NR]** |  |
| 5.1 | | | The importance of treatment adherence | | | | |  | | | | | | | | | | |  | | | | | | |  |  |
| 5.2 | | | The need for a treatment supporter | | | | |  | | | | | | | | | | |  | | | | | | |  |  |
| 5.3 | | | What to do if side-effects occur, they run out of medicines, or need to leave for another area beyond the facility catchment area | | | | |  | | | | | | | | | | |  | | | | | | |  |  |
| 5.4 | | | **[ASK ONLY IF 1.2=YES]**  Review the progress of each TB patient registered for treatment at the facility according to the national guidelines (i.e. For DS-TB Patient, review at months 2 and or 3, 5 and end of treatment and for DR-TB, review monthly till end of treatment) | | | | |  | | | | | | | | | | |  | | | | | | |  |  |
| 5.5 | | | Refer TB patients for appropriate care when necessary | | | | |  | | | | | | | | | | |  | | | | | | |  |  |
| 5.6 | | | Manage contacts according to TB program guidelines | | | | |  | | | | | | | | | | |  | | | | | | |  |  |
| 5.7 | | | Offer HIV testing and counselling to all diagnosed TB clients | | | | |  | | | | | | | | | | |  | | | | | | |  |  |
| 6. | | | ***Drug Regimens* [TB Focal Person]** | | | | | | | | | | | | | | | | | | | | | | | |  |
|  | | | **[ASK ONLY IF 1.2=YES]**  Do staff or personnel initiate and prescribe drug regimens in line with existing national protocol for: | | | | | **Y** | | | | | | | | | | | **N** | | | | | | | **[NR]** |  |
| 6.1 | | | Newly diagnosed patients | | | | |  | | | | | | | | | | |  | | | | | | |  |  |
| 6.2 | | | Re-treatment patients | | | | |  | | | | | | | | | | |  | | | | | | |  |  |
| 6.3 | | | **[ASK ONLY IF 1.6.11 (a)=YES]**  Adult contacts - Preventive treatment for TB infection (INH + Pyridoxine) | | | | |  | | | | | | | | | | |  | | | | | | |  |  |
| 6.4 | | | **[ASK ONLY IF 1.6.11 (a)=YES]**  Child contacts - Preventive treatment for TB infection (INH + Pyridoxine) | | | | |  | | | | | | | | | | |  | | | | | | |  |  |
| 7. | | | ***Management of Children with TB* [TB Focal Person]** | | | | | | | | | | | | | | | | | | | | | | | |  |
| 7.1 | | | **[ASK ONLY IF 1.1.1 & 10.1=YES]**  How are children screened and diagnosed? | | | | | **Yes, Unprompted** | | | | | | | | | | | **Yes, Prompted** | | | | | | | **No** |  |
| 7.1.1 | | | Identify children with presumptive TB | | | | |  | | | | | | | | | | |  | | | | | | |  |  |
| 7.1.2 | | | Refer all children for evaluation to an accessible site | | | | |  | | | | | | | | | | |  | | | | | | |  |  |
| 7.1.3 | | | Identify the child contacts of all smear positive, Xpert MTB/RIF positive, or all pulmonary TB patients | | | | |  | | | | | | | | | | |  | | | | | | |  |  |
| 7.1.4 | | | Use tuberculin skin test (TST) in children under five | | | | |  | | | | | | | | | | |  | | | | | | |  |  |
| 7.1.5 | | | Check the TST induration (reading of tuberculin skin test results) | | | | |  | | | | | | | | | | |  | | | | | | |  |  |
| 7.1.6 | | | Other (specify) | | | | |  | | | | | | | | | | |  | | | | | | |  |  |
| 7.2 | | | **[ASK ONLY IF 1.2.1=YES]**  What kind of care do you provide children? | | | | | **Yes, Unprompted** | | | | | | | | | | | **Yes, Prompted** | | | | | | | **No** |  |
| 7.2.1 | | | **[ASK ONLY IF 1.2.1=YES]**  Initiate drug regimens for children in line with existing national recommendations for treatment of childhood TB for children with active disease | | | | |  | | | | | | | | | | |  | | | | | | |  |  |
| 7.2.2 | | | **[ASK ONLY IF 1.2.1=YES]**  Other (specify) | | | | |  | | | | | | | | | | |  | | | | | | |  |  |
| 8. | | | ***Specimen Management* [Lab Personnel or TB Focal Person]** | | | | | | | | | | | | | | | | | | | | | | | |  |
| 8.1 | | | How is sputum collected? | | | | | **Yes, Unprompted** | | | | | | | | | | | **Yes, Prompted** | | | | | | | **No** |  |
| 8.1.1 | | | Immediately out of bed in the morning (before eating or drinking anything), brush teeth and rinse mouth with water only | | | | |  | | | | | | | | | | |  | | | | | | |  |  |
| 8.1.2 | | | Take deep breath through mouth (breath in and out 3 times) | | | | |  | | | | | | | | | | |  | | | | | | |  |  |
| 8.1.3 | | | Cough up mucous from deep in the chest | | | | |  | | | | | | | | | | |  | | | | | | |  |  |
| 8.1.4 | | | Spit the mucous into special plastic cup or jar – screw lid tightly | | | | |  | | | | | | | | | | |  | | | | | | |  |  |
| 8.1.5 | | | Put the cup or jar into the bag it came in and seal the bag closed | | | | |  | | | | | | | | | | |  | | | | | | |  |  |
| 8.1.6 | | | Put the specimen into the refrigerator until it is returned to the clinic | | | | |  | | | | | | | | | | |  | | | | | | |  |  |
| 8.1.7 | | | Other (specify) | | | | |  | | | | | | | | | | |  | | | | | | |  |  |
|  | | | **[REQUEST COPIES OF EACH GUIDELINE TO BE SIGHTED BEFORE INDICATING “YES”; NONAVAILABILITY SHOULD BE REGARDED AS “NO”]** | | | | | **Y** | | | | | | | | | | | **N** | | | | | | | **[NR]** |  |
| 8.2 | | | Are SOPs for specimen collection available? | | | | |  | | | | | | | | | | |  | | | | | | |  |  |
| 8.3 | | | Does the facility have the contact details of their laboratory? | | | | |  | | | | | | | | | | |  | | | | | | |  |  |
| 8.4 | | | Are the approved laboratory request forms available? | | | | |  | | | | | | | | | | |  | | | | | | |  |  |
| 8.5 | | | Is there an up-to-date specimen dispatch list? | | | | |  | | | | | | | | | | |  | | | | | | |  |  |
| 8.6 | | | Were there any stock-outs of specimen supplies in the past 6 months? | | | | |  | | | | | | | | | | |  | | | | | | |  |  |
| 8.7 | | | Is the laboratory onsite? | | | | |  | | | | | | | | | | |  | | | | | | |  |  |
| 8.7.1 | | | **[ASK ONLY IF 8.7=NO or NR]**  Does specimen transportation to the laboratory occur (within 48 hours) | | | | |  | | | | | | | | | | |  | | | | | | |  |  |
| 8.7.2 | | | **[ASK ONLY IF 8.7=NO or NR]**  Does the facility use a cooler box reserved for transportation of specimens? | | | | |  | | | | | | | | | | |  | | | | | | |  |  |
| 8.2 | | | Are SOPs for specimen collection available? | | | | |  | | | | | | | | | | |  | | | | | | |  |  |
|  | | |  | | | | |  | | | | | | | | | | | | | | | | | | |  |
| 8.7.3 | | | **[ASK ONLY IF 8.7=NO or NR]**  On average, how many days does it take to receive the results at the facility? | | | | | Days ………………  Don’t know …………….…. | | | | | | | | | | | | | | | | | | |  |
|  | | | How would you rate the following on a scale of 1 to 5 where 1=Never, 2=Seldom, 3=Half of the time, 4=Most of the time, and 5=Always? | | | | | **Never** | | **Seldom** | | | | | | | | **Half of the time** | | **Most of the time** | | | | | | **Always** |  |
| 8.8 | | | How often is the facility in compliance in the use of SOPs for specimen collection? | | | | |  | |  | | | | | | | |  | |  | | | | | |  |  |
| 8.9 | | | How often are specimen results returned to the facility in a timely way? | | | | |  | |  | | | | | | | |  | |  | | | | | |  |  |
| 9. | | | ***Adequacy of Infection Prevention Measures* [Infection Control Focal Person or TB Focal Person]** | | | | | | | | | | | | | | | | | | | | | | | |  |
|  | | | I’m going to ask about infection prevention measures and then I’d like to see the supplies used for infection control | | | | | **Y** | | | | | | **N** | | | | | | | | **[NR]** | | | | |  |
| 9.1 | | | A staff member has been designated as an infection prevention and control focal point with specifically articulated duties | | | | |  | | | | | |  | | | | | | | |  | | | | |  |
| 9.2 | | | TB infection prevention and control practices are followed according to national guidelines | | | | |  | | | | | |  | | | | | | | |  | | | | |  |
| 9.3 | | | Patients are routinely asked about cough when entering the facility | | | | |  | | | | | |  | | | | | | | |  | | | | |  |
| 9.4 | | | A system is in place to screen and evaluate staff for TB disease | | | | |  | | | | | |  | | | | | | | |  | | | | |  |
| 9.5 | | | Staff are offered an HIV test annually and offered ART if HIV+ | | | | |  | | | | | |  | | | | | | | |  | | | | |  |
| 9.6 | | | INH (or other preventive therapy) is offered to HIV+ staff | | | | |  | | | | | |  | | | | | | | |  | | | | |  |
|  | | | PLEASE **OBSERVE** TO SEE IF THE FOLLOWING RESOURCES/SUPPLIES USED FOR INFECTION CONTROL ARE AVAILABLE IN THE FACILITY WHERE TB PATIENTS ARE RECEIVING SERVICES ON THE DAY OF ASSESSMENT. **[ASK TO SEE THE ITEMS]** | | | | | **Observed** | | | | | **Reported, not observed** | | | | | | | **Not available** | | | | | **[NR]** | |  |
| 9.7 | | | An updated and approved infection prevention and control plan is available for the facility | | | | |  | | | | |  | | | | | | |  | | | | |  | |  |
| 9.8 | | | A TB infection prevention and control risk assessment is completed at least annually | | | | |  | | | | |  | | | | | | |  | | | | |  | |  |
| 9.9 | | | **[ASK ONLY IF 1.2=YES]**  There is a facility reporting system for all patients diagnosed with TB and referred for treatment (in accordance with national policies) | | | | |  | | | | |  | | | | | | |  | | | | |  | |  |
| 9.10 | | | Cough triage is implemented (patients that are coughing are separated from others and fast-tracked for evaluation) | | | | |  | | | | |  | | | | | | |  | | | | |  | |  |
| 9.11 | | | A cough monitor or other designated person assists with separation and triage of coughing patients | | | | |  | | | | |  | | | | | | |  | | | | |  | |  |
| 9.12 | | | Supplies are available to coughing patients (tissues, masks, etc.) | | | | |  | | | | |  | | | | | | |  | | | | |  | |  |
|  | | |  | | | | |  | | | | |  | | | | | | |  | | | | |  | |  |
| 9.13 | | | Specimens are collected in any of the following designated areas: | | | | | **Observed** | | | | | **Reported, not observed** | | | | | | | **Not available** | | | | | **[NR]** | |  |
| 9.13.1 | | | Outside the service delivery point | | | | |  | | | | |  | | | | | | |  | | | | |  | |  |
| 9.13.2 | | | Away from other patients | | | | |  | | | | |  | | | | | | |  | | | | |  | |  |
| 9. | | | ***Adequacy of Infection Prevention Measures* [Infection Control Focal Person or TB Focal Person]** | | | | | | | | | | | | | | | | | | | | | | | |  |
| 9.13.3 | | | In a well-ventilated area | | | | | |  | | | | | |  | | | | |  | | |  | | | |  |
| 9.14 | | | A confidential log is kept for all staff with presumptive or confirmed TB | | | | | |  | | | | | |  | | | | |  | | |  | | | |  |
| 9.15 | | | Patient waiting areas are outside or have access to fresh air continuously | | | | | |  | | | | | |  | | | | |  | | |  | | | |  |
| 9.16 | | | Surgical masks are available and worn by presumptive and TB patients | | | | | |  | | | | | |  | | | | |  | | |  | | | |  |
|  | | |  | | | | | | **Observed** | | | | | | **Reported, not observed** | | | | | **Not available** | | | **[NR]** | | | |  |
| 9.17 | | | N-95 and FFP2 respirators are readily available for staff | | | | | |  | | | | | |  | | | | |  | | |  | | | |  |
|  | | | **ASK ONLY IF 9.17=YES]** | | | | | | | | | | | | **Y** | | | | | **N** | | | **[NR]** | | | |  |
| 9.17.1 | | | Staff have been trained on proper fit of respirators | | | | | | | | | | | |  | | | | |  | | |  | | | |  |
|  | | | **[ASK ONLY IF 9.17=YES]** | | | | | | | | | **Never** | | | | **Seldom** | | | | **Half of the time** | **Most of the time** | | | | | **Always** |  |
| 9.17.2 | | | How would you rate the use of N-95 and FFP2 respirators by the facility staff on a scale of 1 to 5 where 1=Never, 2=Seldom, 3=Half of the time, 4=Most of the time, and 5=Always? | | | | | | | | |  | | | |  | | | |  |  | | | | |  |  |
|  | | |  | | | | | | | | |  | | | |  | | | |  |  | | | | |  |  |
